# Role of vascular smooth muscle cell clonality in atherosclerosis

**DOI:** 10.3389/fcvm.2023.1273596

**Published:** 2023-11-28

**Authors:** Lingfeng Luo, Changhao Fu, Caitlin F. Bell, Ying Wang, Nicholas J. Leeper

**Affiliations:** ^1^ Division of Vascular Surgery, Department of Surgery, Stanford University School of Medicine, Stanford, CA, United States; ^2^ Stanford Cardiovascular Institute, Stanford, CA, United States; ^3^ Division of Cardiovascular Medicine, Department of Medicine, Stanford University School of Medicine, Stanford, CA, United States; ^4^ Department of Pathology and Laboratory Medicine, University of British Columbia, Vancouver, BC, Canada

**Keywords:** vascular smooth muscle cell, atherosclerosis, clonality, phenotype plasticity, cardiovascular disease

## Abstract

Atherosclerotic cardiovascular disease remains the leading cause of death worldwide. While many cell types contribute to the growing atherosclerotic plaque, the vascular smooth muscle cell (SMC) is a major contributor due in part to its remarkable plasticity and ability to undergo phenotype switching in response to injury. SMCs can migrate into the fibrous cap, presumably stabilizing the plaque, or accumulate within the lesional core, possibly accelerating vascular inflammation. How SMCs expand and react to disease stimuli has been a controversial topic for many decades. While early studies relying on X-chromosome inactivation were inconclusive due to low resolution and sensitivity, recent advances in multi-color lineage tracing models have revitalized the concept that SMCs likely expand in an oligoclonal fashion during atherogenesis. Current efforts are focused on determining whether all SMCs have equal capacity for clonal expansion or if a “stem-like” progenitor cell may exist, and to understand how constituents of the clone decide which phenotype they will ultimately adopt as the disease progresses. Mechanistic studies are also beginning to dissect the processes which confer cells with their overall survival advantage, test whether these properties are attributable to intrinsic features of the expanding clone, and define the role of cross-talk between proliferating SMCs and other plaque constituents such as neighboring macrophages. In this review, we aim to summarize the historical perspectives on SMC clonality, highlight unanswered questions, and identify translational issues which may need to be considered as therapeutics directed against SMC clonality are developed as a novel approach to targeting atherosclerosis.

## Introduction

Despite recent therapeutic advances, cardiovascular disease (CVD) remains the leading cause of death worldwide (1). The most lethal forms of CVD, coronary artery disease and stroke, are both driven by atherosclerosis, a pathological process manifested by the accumulation of plaques containing fatty deposits and cellular debris in the vessel wall. Numerous cell types, including dysfunctional endothelial cells, foamy macrophages, activated medial cells, and dysfunctional phagocytes all contribute to the pathogenesis of atherosclerosis (2). Amongst these, the vascular smooth muscle cell (SMC) is increasingly recognized as playing a major role in disease initiation and progression (3). Under physiological conditions, quiescent SMCs in the medial layer of arteries maintain vascular tone, regulate blood pressure, and secrete extracellular matrix to provide physical support and structural integrity to the vessel wall. However, SMCs are not forced to maintain this “contractile” phenotype and demonstrate a remarkable amount of plasticity (4–6) in response to vascular injury or pathological cues such as hyperlipidemia, hyperglycemia, and inflammation. While “de-differentiation” of SMCs was classically tracked by the loss of so-called SMC-specific markers such as Myh11 (4, 5, 7, 8), it is now clear that many molecules previously considered lineage-restricted are in fact quite promiscuous and can be expressed by a wide variety of cells in response to environmental cues (9–11). For example, cultured SMCs exposed to oxidized lipids are now known to upregulate markers traditionally associated with macrophages, such as CD68 (12, 13). Accordingly, early studies which relied on immunohistochemistry to define the origin of cells that constitute the atherosclerotic plaque likely misidentified many lesional cells and confused our understanding of how the disease develops. The emergence of murine lineage-tracing techniques has allowed investigators to definitively trace the natural history of SMCs during atherogenesis and confirmed that their *in vivo* plasticity is more remarkable than ever suspected (14–16). For example, it is now understood that classic SMC markers can be expressed by cells from a non-SMC lineage, i.e., endothelial cells undergoing endothelial-to-mesenchymal transition (9), and that progeny of SMCs not only lose their “cell-specific” markers during atherogenesis (5, 15) but also gain markers associated with a wide range of other cell types, including “macrophage-like” cells (5, 14). Phenotype-switching is now a major focus on many groups, and the genes that govern these transitions have begun to be mapped (5, 6, 15–17). The phenotypic plasticity of vascular SMCs has been extensively reviewed elsewhere (3, 18), and will not be the focus of this review.

In the context of atherosclerosis, SMC plasticity is considered to be a major determinant of plaque stability (19, 20). Plaques with less lipid content and thicker fibrous caps comprised of layers of SMCs are generally considered stable and at lower risk of rupture and adverse clinical outcomes. Those with larger necrotic cores and thinner caps with reduced SMC content are considered unstable or vulnerable. Therefore, SMCs have been historically considered protective and drivers of plaque stability. However, recent lineage-tracing studies have revealed that SMCs are not restricted to the fibrous cap, but also contribute significantly to the cells within the plaque, itself (15, 21, 22). Knowledge is still limited regarding the mechanisms by which SMCs choose to adopt either a collagen-producing phenotype (which is thought to be anti-atherogenic) or a de-differentiated hyperproliferative phenotype (which is thought to be pro-atherogenic), though several genetically-validated factors have been recently identified (6, 23–26). Where SMCs in the plaque originate from and how those cells expand inside the vessel wall are questions that have been pursued by vascular biologists for decades (14, 15, 21, 27, 28). Elucidation of the biology underlying SMC transitions will not only help us understand the fundamental pathology of CVD, but also promises to identify methods to enhance plaque stability and ameliorate atherosclerosis.

Amongst all of the cell-fate decisions the vascular SMC is capable of making, one particular form of phenotype switching has recently (re)captured the attention of the field: clonal expansion. Clonal expansion is the process by which a specific subpopulation of cells proliferates and accumulates as the progeny of a single parent cell. This concept was originally advanced to describe how the adaptive immune system worked, wherein certain lymphocyte precursor cells (especially B cells and T cells) recognizing specific antigen(s) proliferate into expanded clones (29, 30). Later, a similar concept was adopted in the oncology field (31, 32), where tumors grow due to cells acquiring genetic mutations and expanding in a clonal fashion. More recently, clonal expansion has been observed in a range of vascular cell types, including endothelial cells (33) and myeloid cells (termed “clonal hematopoiesis of indeterminate potential” or CHIP) (34, 35), and their critical role in CVD has been elegantly reviewed by others (36). However, in the specific context of atherosclerosis, there is increasing experimental evidence (at least in mice) that clonal SMC expansion may play a dominant role, where they significantly contribute to the cellular mass within a developing plaque (15, 37). In this brief review, we will summarize the published studies on vascular SMC clonality, discuss the gaps in our knowledge about how SMCs regulate plaque formation, and explore the translational potential of targeting SMCs in atherosclerosis.

## Evidence for SMC clonality in the tunica media

Early studies relied on X-chromosome inactivation to determine if SMCs expand clonally in arterial tissue. During embryonic development, female cells have one of their X chromosomes (paternal or maternal) randomly and permanently inactivated in a process known as lyonization. Pioneering work conducted by Benditt & Benditt performed zymograms to measure the enzymatic activity of the X-linked gene, glucose-6-phosphate dehydrogenase (G6PD), on vascular samples. These studies revealed that normal aortic and iliac arterial walls are composed of both G6PD^+^ and G6PD^−^ cell types (27). This suggested a polyclonal pattern of cell expansion, as a single isotype would be expected if all vascular cells were derived from a monoclonal origin. However, this study was limited by a lack of efficient methods to distinguish specific cell types or trace their cellular origins. Later, using more sensitive polymerase chain reaction (PCR) assays of X-linked androgen receptor genes applied to micro-dissected arterial SMCs (defined as regions staining positive for an SMC marker), Murry et al. observed that the majority of (22) samples contained both paternal and maternal patterns of X inactivation, thus confirming the polyclonality of medial SMCs in humans (38, 39). This polyclonal pattern was further validated by Jacobsen and his colleagues (22). Using chimeras of *eGFP*^+^ *Apoe*^−/−^ and *Apoe*^−/−^ mouse embryos, they found that unlike the large patch size identified by earlier human studies using X-inactivation, mouse aortic media exhibited small patches of SMCs with a single color (*eGFP*^+^ or *eGFP*^−^) which were interspersed with cells of the other color (22). With the assistance of SMC-specific markers, these studies were able to locate SMCs that maintained a high level of marker expression, but there was still a lack of resolution (only being able to view cell patches) and sensitivity (being affected by phenotypic modulation and unable to trace cell lineage).

**Figure 1:**
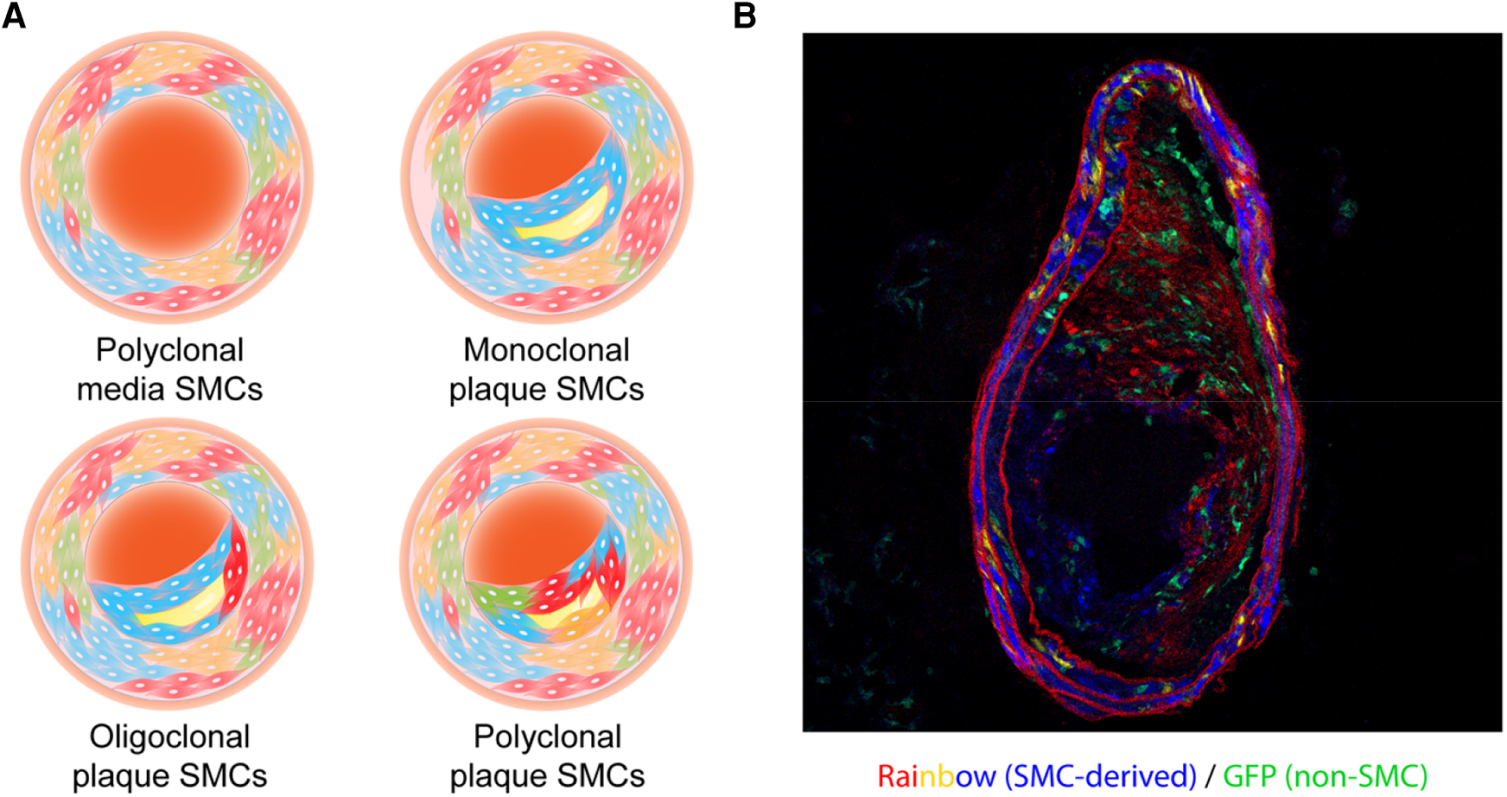
(**A**) illustration of the clonality patterns that have been observed in various studies. The vascular smooth muscle cells (SMCs) in the tunica media exist in a polyclonal manner, while plaque SMCs can show a monoclonal, oligoclonal, or polyclonal pattern depending on environmental cues. (**B**) A representative cross-sectional image of an atherosclerotic vessel from a male *Myh11-CreER^T2^*, ROSA26R- Rainbow, *Apoe*^−/−^ mouse. This mouse carried a “multi-color” Rainbow reporter that constitutively expressing GFP. After being injected with tamoxifen to activate Cre recombinase in Myh11-expressing cells, this mouse was fed with a high-fat diet. These Myh11-expressing cells and their progeny were randomly labeled with one of the Rainbow colors (Cerulean, mOrange, or mCherry).

The subsequent development of multi-color lineage tracing systems allowed researchers to trace cellular origin more precisely. One of the systems generated to specifically study clonality, the ROSA26R-Rainbow Cre reporter, induces individual cells to randomly and permanently express one of three fluorophores (Cerulean, mOrange, or mCherry) in response to Cre-induced recombination. The labeled cell and its progeny will continue to express the same color, regardless of phenotype switching or de-differentiation, permitting high-fidelity tracing (including all daughter cells) (40). Using a ubiquitous ROSA26R-Rainbow Cre reporter, Misra et al. gave their mice a tamoxifen pulse (150 µg) at embryonic day E5.25 to induce recombination, allowing them to observe an intermixed pattern with three colors in the descending aorta at a later embryonic stage (E12.5) or in adulthood (21), which means around E5.25, there were multiple smooth muscle progenitor cells that eventually generated SMCs in the media of the descending aorta. This team also validated their results using an X-linked *GFP* transgenic mouse line where they observed a mixture of *GFP*^+^ and *GFP*^−^ in both the embryonic and adult descending aorta (21). These results clearly suggest the polyclonality of medial SMCs during development (Figure 1A, Table 1).

**Table 1:** Summary of studies on SMC clonality.

Species	Sex	Locations	Methodology	Clonality	Conclusions	Limitations	References
*Intact arteries or the tunica media of atherosclerotic lesions							
Human	Female	Aorta and iliac arteries	Microdissection, zymograms for the X-linked G6PD	Polyclonal	Media samples showed mixed X inactivation patterns	- Limited resolution and sensitivity	(27)
Human	Female	Aorta	Microdissection, zymograms for the X-linked G6PD	Polyclonal	Media samples showed balanced X inactivation patterns	- Limited resolution and sensitivity	(42)
Human	Female	Aorta and coronary arteries	Microdissection, PCR for the X-linked androgen receptor gene	Polyclonal	Most media samples showed balanced X inactivation patterns	- Limited resolution and sensitivity	(38, 39)
Mouse	N/A	Aorta	Chimera mice of *eGFP*^+^*Apoe*^−/−^ and *Apoe*^−/−^ mouse	Polyclonal	Media showed small patches with the same color	- Low sensitivity (could not distinguish cell types)	(22)
Mouse	Male (ROSA26R-*CreER^T2^*) Female (X-linked GFP)	Aorta	The ubiquitous ROSA26R-*CreER^T2^* mice injected with tamoxifen at embryonic day E5.25-12.5; X-linked GFP transgenic female mice	Polyclonal	Media cells of embryos and adults showed mixed colors.		(21)
*Atherosclerotic plaques							
Human	Female	Aorta and iliac arteries	Microdissection, zymograms for the X-linked G6PD	Monoclonal	Fibrous cap cells showed predominantly one X inactivation pattern	- Limited resolution and sensitivity	(27, 41)
Swine	Male	Aorta	Tritiated thymidine (3HTdR) injection before hypercholesterolemic diet for 30–60 days	Polyclonal	All the atheorsclerotic lesions were polyclonal in origin	- Limited resolution and sensitivity	(28)
Human	Female	Aorta	Microdissection, zymograms for the X-linked G6PD	Polyclonal	All subregions of most lesions showed mixed X inactivation patterns	- Limited resolution and sensitivity	(42)
Human	Female	Aorta and coronary arteries	Microdissection, PCR for the X-linked androgen receptor gene	Undetermined	Most plaque SMCs showed a single pattern of X inactivation; the pre-existing patches in normal arteries were large	- Low resolution; - Lack of track of cellular origins	(38, 39)
Mouse	N/A	Aorta	*Sm22a-CreER^T2^*, ROSA26R-Confetti, *Apoe*^−/−^ mice were induced with tamoxifen and fed a HFD for 12 weeks	Undetermined	Clonally grown SMC-derived plaques showed either red or green color	- Limited sample size and resolution	(14)
Mouse	N/A	Aorta and carotid arteries	*Myh11- CreER^T2^*, ROSA26R-Confetti, *Apoe*^−/−^ mice were induced with tamoxifen and fed an HFD for 16–19 weeks	Oligoclonal	The majority of SMCs in a plaque came from one or two cells		(15)
Mouse	N/A	Aorta	Chimera mice of *eGFP *^+ ^*Apoe*^−/−^ and *Apoe*^−/−^ mouse; *Myh11- CreER^T2^*, ROSA26R-Confetti mice infected with rAAV8-D377Y-PCSK9 and fed an HFD for 12, 24, or 36 weeks	Oligoclonal	The majority of SMCs in a plaque came from one single cell, but small amount of plaques had SMCs from two origins		(22)
Mouse	Both	Aortic roots and brachiocephalic arteries	*Myh11-CreER^T2^* or *Acta2-CreER^T2^*, ROSA26R-Rainbow, *Apoe*^−/−^ mice were induced with tamoxifen and fed an HFD for 6, 12, or 16 weeks	Monoclonal	The majority of SMCs in a plaque came from one progenitor; Itgb3 in bone marrow-derived cells regulated SMC clonality in atherosclerotic plaques		(21)
Mouse	Male	Brachiocephalic arteries	*Myh11-CreER^T2^*, ROSA26R-Rainbow, *Apoe*^−/−^ mice were induced with tamoxifen and fed an HFD for 6–32 weeks	Oligoclonal/polyclonal	Anti-CD47 treatment shifted plaque SMCs from monoclonal to polyclonal		(44)
Mouse	Male	Aortic roots and brachiocephalic arteries	3- or 18-month-old, *Myh11-CreER^T2^* or *Csf1r-Mer-iCre-Mer*, ROSA26R-Rainbow mice infected with rAAV8-D377Y-PCSK9 and fed an HFD; Aged or *Itgb3*^−/−^ BMT to *Myh11-CreER^T2^*, ROSA26R-Rainbow, *Ldlr*^−/−^ mice fed an HFD	Monoclonal/polyclonal	Aged bone marrow shifted plaque SMCs from monoclonal to polyclonal		(43)

## Evidence for SMC clonality in atherosclerosis

The earliest exploration of SMC clonality in plaques was also pioneered by Earl Benditt and his son nearly 50 years ago (25). Using X-inactivation analysis of G6PD zymograms, the hypothesis that lesions could be driven by an SMC neoplasm was developed as researchers reported that fibrous-capped plaques were predominantly composed of cells with a single isotype (27, 41). However, using the same method in a larger sample cohort, Thomas et al. showed that while cells on the top layer of a plaque tended to have a monoclonal component (with a lower percentage of monoclonality than Benditt & Benditt's results), the entire lesions were intermixed with both G6PD-A and G6PD-B patterns, indicating polyclonality (42). Moreover, Thomas et al. injected tritiated thymidine (^3^HTdR) into swine and fed the animals a hypercholesterolemic diet for 30–60 days to trace the proliferation of vascular cells during early lesion development in that species (28). Similar to studies on human samples, they found most of the pig plaques consisted of cells from mixed origins (28). As discussed earlier, one of the main limitations of these pioneering studies was the lack of a method to definitively trace cells over time, especially given our current understanding of phenotype switching and the danger of relying on “cell-specific” marker expression to identify the origin of a given cell. Furthermore, unlike the media which is mainly comprised by SMCs, atherosclerotic plaques consist of multiple cell types, including but not limited to SMCs, macrophages, endothelial cells, T cells, B cells, and dendritic cells. Each of these cell types could theoretically be recruited into the developing plaque independently, thus diluting any signal that might be present from a clonally-expanding SMC.

Subsequent higher-resolution, PCR-based analyses of the human androgen receptor locus revealed that the majority of plaque SMCs (as defined by the expression of SMC markers) demonstrated a single X-linked pattern, suggesting those cells were potentially clonal (39). However, due to the large size of pre-existing X-inactivation patches in arteries with diffuse intimal thickening, these studies could not clearly distinguish whether plaque SMCs are exclusively monoclonal (38). For example, plaque SMCs may arise from a single progenitor cell within a given patch, or arise from a subpopulation of SMCs derived from the same pre-existing clone. Though the use of SMC markers helped improve confidence around cellular specificity in these studies, the plasticity of SMCs during atherogenesis remained a major issue that prohibited the definitive identification of each cell's origin. For example, SMC marker expression is now known to be greatly reduced in the necrotic core during atherogenesis (14, 15), and the source of those cells could not have been conclusively defined at the time these studies were conducted (38, 39).

The first conclusive evidence that plaque SMCs undergo clonal expansion, at least in mice, came from Feil and colleagues who used a much more rigorous ROSA26R-confetti lineage-tracing system (14). Similar to the ROSA26R-rainbow system described above, individual cells can be labeled stochastically with one of the four fluorophores including cytoplasmic red, cytoplasmic yellow, membrane-bound blue, and nuclear green, upon Cre recombination. Feil and colleagues used atheroprone *Apoe*^−/−^ mice carrying both ROSA26R-confetti multicolor Cre reporter and SM22α-driven tamoxifen-inducible Cre recombinase, CreER^T2^, to trace individual SMCs (14). These mice were subjected to a tamoxifen pulse when 10 weeks old and fed a high-fat diet (HFD) for the ensuing 12 weeks to induce plaque formation. Plaque SMCs were observed to predominantly show a single color suggesting oligoclonality, though sample size and resolution made it difficult to determine whether these SMCs came from one or a handful of cells. Using *Myh11-CreER^T2^* ROSA26R-Confetti mice fed an HFD, Chappell et al. found that though the plaques were not exclusively monoclonal (only 52% contained a single color), SMCs in the monochromatic regions of plaques appeared to derive from a single progenitor (15). A similar oligoclonal pattern of SMC expansion was also observed in the neointima of the carotid artery ligation model (15).

Using chimeras of *eGFP*^+^ *Apoe*^−/−^ and *Apoe*^−/−^ mouse embryos, Jacobsen et al. observed larger lesional SMC patch sizes than in the media indicating oligo-expansion of SMCs in plaques (22). In the same study, the authors also used *Myh11-CreER^T2^* Confetti mice infected with rAAV8-D377Y-PCSK9 and fed an HFD to trace medial SMCs during plaque formation (22). They found most of the plaque SMC populations expressed a single color, further indicating that SMCs within a plaque came from a single cellular origin, though in rare cases overlap between 2 patches with different colors was observed. Notably, mice with longer HFD exposure (36 weeks) tended to have a higher chance of developing SMC patches with mixed origins than mice fed HFD for a shorter duration (12 or 24 weeks) (22). Both Chappell and Jacobsen found SMCs in the fibrous cap were positive for SMC-specific markers, but cells inside the core area had lost expression of those genes. In contrast, in the non-atherosclerotic carotid ligation model, neointimal SMCs still retained expression of those markers (15).

In one of the first studies pursuing the molecular mechanisms that might promote the observed clonal expansion of SMCs in atherosclerosis, Misra et al. conducted a series of elegant experiments in mice containing the ROSA26R-Rainbow system driven by *Myh11- CreER^T2^* or *Acta2-CreER^T2^* on the *Apoe*^−/−^ genetic background (21). These mice were induced with tamoxifen and then fed an HFD for 6, 12, or 16 weeks to promote lesion development. The researchers reported that all of the plaque SMCs they analyzed were labeled with a single Rainbow color in both the aortic root and the brachiocephalic artery (21). Intriguingly, they found that deletion of *Itgb3* (encoding Integrin Subunit Beta 3) in bone marrow-derived cells stimulated medial SMC transdifferentiation and shifted the monoclonal pattern of plaque SMCs towards a polyclonal status. This was the first evidence that disturbance of one gene could regulate the clonality of SMCs during plaque development. This study also led the investigators to hypothesize that medial SMCs first expand into the fibrous cap and from there invade the core region of the plaque, rather than migrate into the two regions separately.

A recent follow-up study by the same group reported that bone marrow from aged mice—which express less *Tet2,* have epigenetically silenced *Itgb3*, and have increased TNF signaling—stimulates polyclonal SMC expansion in atherosclerotic lesions, contrary to the monoclonality of plaque SMCs in young mice (43). Anti-TNF antibody treatment restored plaque SMC monoclonality and decreased lesion size caused by *Itgb3*^−/−^ or aged bone marrow (43), shedding light on the translational potential of targeting TNF signaling and its relationship to SMC clonality during atherogenesis. Their work was the first to highlight the critical importance of SMC-macrophage crosstalk during clonal expansion and underscores the fact that both cell autonomous and non-autonomous factors may dictate the emergence of individual SMC clones as the lesion progresses, especially with aging.

Using a similar ROSA26R-Rainbow system driven by *Myh11- CreER^T2^* in *Apoe*^−/−^ mice, our group observed an oligoclonal pattern of SMCs in brachiocephalic artery plaques (Figure 1B), and described how they might exacerbate vascular inflammation once clonally expanding in the lesion (44). We found that Sca1^+^ SMCs in and near the necrotic core expressed high levels of complement component C3 which might feed forward to accelerate cellular proliferation, thus contributing to the relative growth advantage of the dominant clone. We also hypothesized that the production of anaphylatoxins downstream in the complement cascade could exacerbate inflammation and lesional vulnerability, and provided data suggesting that there may be a phagocytic defect that prevents macrophages from identifying and removing these pathological cells. As discussed below, restoration of efferocytic machinery in the plaque via blockade of CD47 (another factor related to TNF-α signaling) led to the removal of the dominant clone, a restoration of polyclonality, and smaller lesions.

In summary, the totality of these increasingly sophisticated lineage tracing studies has firmly consolidated the consensus that there is mono- or oligo-clonal expansion of SMCs during atherogenesis (Figure 1A,B; Table 1), at least in mice. While methods available for confirmation in human samples continue to lag behind those used in murine studies, some data argue that a similar phenomenon is present in subjects with coronary artery disease (27, 44, 45). One important caveat related to future mechanistic studies is to apply caution when interpreting bone marrow transplantation data, given a recent study conducted by Newman et al. which surprisingly showed that irradiation can profoundly impact smooth muscle expansion in certain atheroprone arterial beds (46).

## Unresolved questions related to SMC clonality

### Who starts the party? Is there such a thing as an “atherosclerotic stem cell”?

The contribution of medial cells to the neointima during atherogenesis or in response to vascular injury has been recognized for decades. Beginning in the 1980s, Clowes et al. infused rats with tritiated thymidine (^3^HTdR) after carotid balloon surgery and found a mixture of proliferative and non-dividing cells derived from the media (47), suggesting a role for direct migration in addition to local proliferation. Subsequent lineage-tracing studies with atheroprone mice have repeatedly demonstrated the contribution of medial SMCs to the plaque (5, 14, 15, 21). Unlike findings in the carotid balloon injury model, which results in endothelium denudation, SMC clonal expansion appears not to result from the direct migration of medial cells in murine atherosclerotic models (15). Because endothelial cell cross-talk and restraint from the internal elastic lamina are thought to affect SMC phenotype modulation and proliferation (48, 49), one can speculate that mechanical damage to the vessel may alter the capacity for SMCs to invade into the growing lesion. In a carotid ligation model that does not directly remove endothelial cells, SMC migration was found to have a trivial contribution to neointima formation (15). Additional studies of neointimal SMC clonality in endothelial denudation models such as carotid balloon injury or femoral artery wire injury should shed light on these issues. Nevertheless, current evidence indicates that in the case of murine atherosclerosis models, SMC proliferation and oligoclonality dominate the SMC population within the plaque.

**Figure 2:**
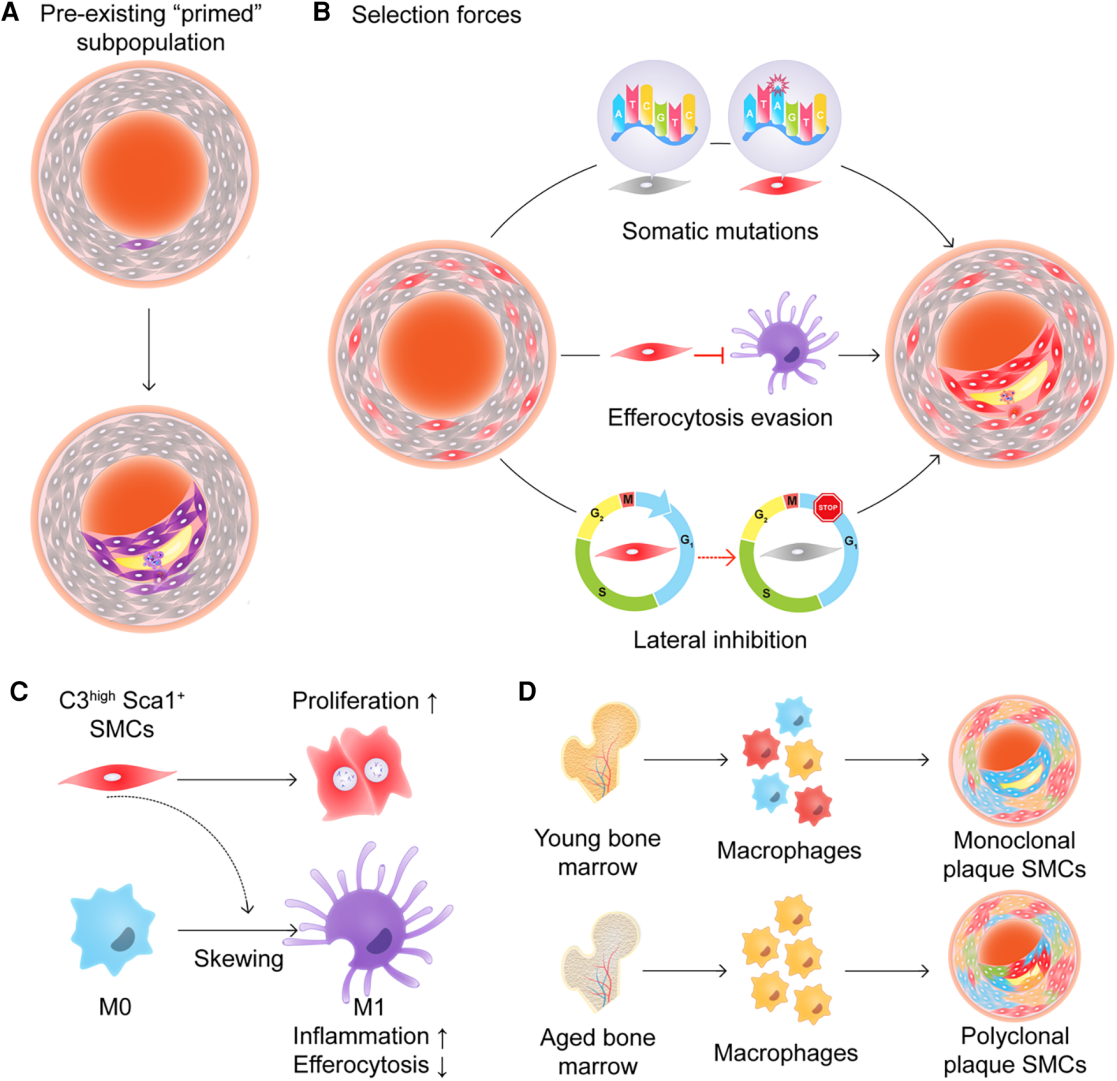
(**A**) and (**B**) Two models of how smooth muscle cells (SMCs) might expand in an atherosclerotic plaque. (**A**) A subpopulation of “primed” progenitors exists in the vessel wall that are more prone to expand than the rest of the SMCs. (**B**) All medial SMCs have expansion potential, but a small subset of them is “selected” due to some type of survival advantage including somatic mutations, efferocytosis evasion, and/or lateral inhibition. (**C**) C3^high^ Sca1^+^ SMCs promote cell proliferation in an autocrine manner and skew macrophages toward a pro-inflammatory phenotype with less efficient efferocytic abilities. (**D**) Aged bone marrow-derived myeloid cells shift plaque SMCs from monoclonal expansion to polyclonal expansion.

The major question, however, is which cell initiates clonal SMC expansion. At its most fundamental level, one can argue that SMC clonality must occur via one of two mechanisms, which are not necessarily mutually exclusive. The first option is that there is a pool of primed progenitors that are more prone to expand than other SMCs (Figure 2A). The second option is that all medial SMCs have expansion potential, but a small subset of them are “selected” due to some type of survival advantage (Figure 2B). Evidence which can be viewed as supportive of the pre-existing progenitor hypothesis can be found in multiple studies (44, 50, 51). For example, Dobnikar et al. used Stem Cell Antigen-1 (Sca1)-GFP transgenic mice and *Myh11- CreER^T2^*-EYFP or -Confetti reporter systems, and identified a rare population of Sca1^+^ medial SMCs (0.2%–1.6% of overall media cells) in healthy vessels which may mark SMCs undergoing phenotypic modulation (50). This subset of cells shared the expression signature of SMCs in atherosclerotic lesions, but was distinct from Sca1^−^ medial SMCs. These pre-existing SMCs had lower expression levels of conventional SMC markers and upregulated genes related to proliferation and inflammation. Though clonal SMC proliferation was observed at low frequency and proliferation capacity was not limited to Sca1^+^ SMCs, it appeared that Sca1^+^ SMCs were more prone to respond to injury and inflammation thus potentially driving oligoclonal expansion (51). Interestingly, both Jørgensen's (50, 51) group and our team (44) observed Sca1 to be highly expressed in SMCs of the plaque core, but not in the fibrous cap, and found the expression of Sca1 to gradually increase during lesion progression (while the dominant clone expands).

Other groups have not observed Sca1^+^ cells in the media before the plaque begins to develop. Wang et al. and Tang et al. used Sca1 lineage tracing mice and found that Sca1 expression was predominantly restricted to the endothelium and adventitia of healthy vessels, and that Sca1^+^ cells did not significantly contribute to expanded SMCs in atherosclerotic lesions (52) or the neointima after wire injury (53). Only in an anastomosis model marked by severe vascular injury were Sca1^+^ adventitial cells found to invade and contribute to the media during tissue repair (53). The apparent discrepancy might partially arise from differences in Cre recombination efficiency due to the differences in tamoxifen dose (51). In addition, it is possible that a small number of Sca1^−^ medial SMCs gain Sca1-positivity early in their response to atherogenic stimuli or injury, including dyslipidemia which occurs in *Apoe*^−/−^ mice even before starting the Western diet (54). Therefore, dual-reporter mice capable of tracing both SMC makers and Sca1, as well as SMC-specific Sca1-conditional knockout mice, are needed to resolve the inconsistencies in the literature. Regardless of whether Sca1 is definitively present in a subset of quiescent medial cells, SMCs clearly have the capacity to upregulate Sca1 during clonal expansion, and this marker may identify cells poised to respond to inflammatory signals.

On the other hand, it is possible that there are no specialized, pre-existing “atherosclerotic stem cells”, and that any medial SMC has the potential to expand and contribute to clonal expansion. Worssam et al. used single cell RNA-sequencing and confocal microscopy to argue that the ability to proliferate may be a general, cell-intrinsic feature of SMCs, and that Sca1 merely is a marker of cells that are committed to phenotype switching and proliferating in response to injurious stimuli (51). If it eventually is proven that all SMCs have equal potential for clonal expansion, mechanistic work will have to determine how these cells become “selected” and how they “outcompete” their neighbors. Hypotheses under consideration include the acquisition of a simple proliferative advantage (44, 51), the development of an ability to escape immune surveillance (44), or the active suppression of neighboring SMCs (21). Nevertheless, it is now consolidated that plaque Sca1^+^ SMCs de-differentiate, have a higher proliferation rate (51), and express higher levels of inflammatory complement components (44) than their counterparts. Future work needs to address how these cells initially respond to cellular or environmental cues, and if inciting events are distinct from those that maintain continued expansion. It will also be interesting to map the sequence of events that change throughout atherogenesis, so we can understand the temporal kinetics of phenotypic modulation, migration from the media, and clonal expansion in the plaque. Studies that combine lineage-tracing systems, single-cell RNA sequencing, and spatial ‘omics technologies might allow for the generation of a map of the initiating steps that permit clonal SMC expansion (6, 44, 51, 55, 56).

### What genetic or environmental cues permit continued clonal expansion?

Benditt & Benditt speculated that the underlying force driving SMC clonality could be somatic mutation resulting from chemical mutagens or viruses (27). Though there has been little direct evidence supporting this hypothesis to date, advances in sequencing technologies (57, 58) will allow investigators to determine if SMCs accumulate mutations that confer a selective growth advantage, as has been seen in the expansion of subpopulations of cancer cells and hematopoietic cells (32, 34). The recent discovery of clonal hematopoiesis of indeterminate potential (CHIP) has proven that acquired mutations in myeloid lineage cells are definitively associated with human atherosclerosis (59). It is interesting to consider the possibility that similar mechanisms that enhance proliferation or suppress programmed cell death may be present in medial cells, as well.

Epigenetic regulation is also known to modulate SMC phenotype (24). Modifications on histones such as H3K9 (60) and H3K4 (61) have been reported to alter SMC inflammation and plasticity. The histone modulator, TET2, is indispensable to maintaining the conventional contractile phenotype of SMCs (61–63), and Liu et al. reported that it functions by modifying H3K4me2 (61). Our group recently performed bulk ATAC sequencing and found that SMCs undergo widespread alterations in chromatin accessibility during atherosclerosis development, with around 25% of accessible regions being perturbed (24). This shift in accessibility pattern was associated with the down-regulation of ATF3, enhanced expression of the clonality-related factor, C3, and activation of the inflammatory phenotype of SMCs.

Apart from intrinsic genetic or epigenetic alterations, the selection of certain SMC clones might be driven by cell-cell crosstalk. The first consideration is that the dominant clone may not only out-compete neighboring cells, but may also actively suppress other SMCs. The concept of lateral inhibition is well described during development (64, 65), and there may be paracrine interactions between SMCs that suppress the emergence of additional clones. The second consideration is that communication with other vascular cells may determine whether an SMC is permitted to clonally expand. For example, we previously found that polarized M1 macrophages in the plaque lose the ability to sense and clear inflamed SMCs, even though they are heavily opsonized and marked for removal. In our model, we proposed that Sca1^+^ SMCs proliferate and promote inflammation due to their production of C3-dependent anaphylatoxins, but escape immune surveillance due to the general defect in efferocytosis which is known to occur during atherogenesis (Figure 2C) (44, 66–70). Additional evidence that SMC-macrophage crosstalk directly dictates SMC clonality was also provided by Kabir and colleagues (21, 43). They found that reduced TET2 expression in aged bone marrow was associated with epigenetic downregulation of *Itgb3* expression and enhanced TNF signaling, and that the transplantation of these macrophages was sufficient to shift the pattern of SMC expansion pattern from a monoclonal to polyclonal status (Figure 2D). As mentioned above, TET2 is one of the most frequently mutated genes in CHIP (34), suggesting a possible link between the clonal expansion of hematopoietic lineage cells and clonal SMC expansion. Overall, these studies indicate that cell-cell communication, whether between SMCs or with other intraplaque cells, may determine whether a SMC which begins to proliferate is ultimately capable of evolving into a dominant clone within the plaque.

### Do developmental origins or anatomic differences influence SMC clonality?

Anatomical considerations may also influence how SMCs colonize lesions. During embryonic development, vascular SMCs arise from various sources, as has been extensively reviewed elsewhere (71, 72). Briefly, SMCs derived from the second heart field comprise the entire media of the aortic sinus and the outer media layer of the ascending aorta, while SMCs derived from the cardiac neural crest comprise the inner media layer of the ascending aorta, the proximal portion of the aortic arch, the brachiocephalic artery, and the carotid arteries (71, 73). The varying regional susceptibility of arteries to vascular diseases has been previously reported (74–76), and Dobnikar et al. provided evidence that SMCs from different developmental origins have distinct transcriptional profiles (50). Therefore, there might be differences in how SMCs clonally expand i.e., in the aortic root vs. in the brachiocephalic artery. Beyond variability attributable to embryological origin, future studies should also investigate the impact of perturbed flow and shear stress on SMC expansion, given the hypotheses that overlying endothelial cells may influence the behavior of the expanding clone (22). It is known that an eccentric lipid core can redistribute circumferential stress to plaque shoulders (77). *In vitro* evidence supports the concept that pathological stretch could induce SMC phenotypic modulation and proliferation (78). Since mechanical forces affect cell competition (79, 80), it will be intriguing to examine how that influences the initiation and progression of SMC clonal expansion. Finally, differences across species and at different ages must be carefully mapped (43). Currently, there are few methods available to trace human SMCs *in vivo (*44, 45, 81), as can be accomplished in mice with indelible fluorescent lineage-tracing systems (14, 40). As the ability to map cell fate with methods based on mitochondrial DNA variant sequencing evolves (57), the discrepancies between mouse and human results described above can be reconciled.

## Imagining the therapeutic potential of targeting clonal SMC expansion

As our understanding of the molecular mechanisms underlying the initiation and maintenance of SMC expansion increases, so too does the likelihood that these processes can one day be targeted for therapeutic purposes. However, several questions regarding the timing of intervention, ideal approach, and theoretical risks and benefits of suppressing this phenomenon merit consideration. For example, should we target the initial SMC activation step or aim to suppress expansion once the cap has been bolstered? Should we aim to remove all dividing SMCs, or will there be opportunities for therapeutic biasing of cells away from an inflammatory state towards one which may have plaque-stabilizing effects? Do we need to shift our focus from the SMC itself to those neighboring macrophages which may be responsible for inappropriately permitting unfettered cell growth? Is there a point late in lesion development where clonal expansion actually becomes a protective mechanism we may wish to encourage?

For example, excessively proliferating SMCs can undergo replicative senescence, as has been observed in the fibrous caps of human atherosclerotic lesions (82, 83). In mice, bypassing senescence by overexpressing TRF2 has been shown to increase cap thickness and stability (84). Given the enhanced growth kinetics of clonally expanding SMCs, it is possible that those cells are more vulnerable to replicative senescence than quiescent medial SMCs. The selective removal of senescent foamy cells has been reported to stabilize the fibrous cap (85). Clearance of senescent cells has also been found to suppress SMC phenotype switching and migration (86). It will be interesting to see if SMC-specific removal of intraplaque senescent cells protects from plaque instability and rupture. Because clonally expanded Sca1^+^ cells express factors that drive the formation of the pro-inflammatory membrane attack complex (44), interventions that induce their removal, such as by targeting the pro-efferocytic CD47-SIRPα axis (66, 67), may prove useful for the clearance of necrotic tissue in the plaque core. However, consideration should be given to the hypothesis that non-selectively targeting SMC clones in the plaque could be a double-edged sword. While limiting the expansion of SMCs in the core region may be beneficial, compromising the proliferative capacity of SMCs in the cap might be detrimental to plaque stability, especially given evidence that core SMCs arise from clonal cells after they have migrated to the cap (21). SMC apoptosis is another known factor contributing to plaque vulnerability (87). Previously, we identified gene *CDKN2B* that might impact p53-dependent apoptosis of SMCs and it was at the chromosome 9p21.3 region that was associated with several vascular diseases including atherosclerosis (88). It will be interesting to study how the balance between SMC clonal expansion and apoptosis affects plaque development and stability. Furthermore, scRNA-seq studies have demonstrated remarkable intrinsic heterogeneity amongst lesional SMCs (44, 50). We may discover methods which target specific subpopulations derived from the “mother” clone, or identify ways to coax them away from a harmful phenotype [e.g., SMC-derived macrophage-like cells (5)], and back towards one that will produce extracellular matrix and prevent plaque erosion. Finally, it remains to be elucidated whether monoclonality or polyclonality is more desirable, and if its role evolves based on the age of the subject and severity of his or her disease. Kabir et al. found *Itgb3* knockout in aged bone marrow or anti-TNFα treatment decreased lesion size while shifting SMCs toward a monoclonal status (43). We found that anti-CD47 treatment decreased plaque size (66) but shifted the monoclonal pattern towards one of stochastic expansion (44). The discrepancies in the direction of the clonal pattern might arise from age differences between these studies. We used much younger mice (6–32 weeks) while the *Itgb3* study included significantly older animals (18 months). It is possible that in youth, the phagocytic clearance of a dominant SMC clone with a pro-inflammatory phenotype can ameliorate disease progression, while in aged mice, TNF*α* secreted by TET2-down-regulated macrophages may stimulate the expansion of secondary clones which promote plaque enlargement. These and other considerations hint at the complexity and potential pitfalls associated with targeting the clonally expanding SMC and highlight the fact their physiology is likely context-dependent.

## Conclusions

Vascular SMCs are fundamental players in atherogenesis due to their competing roles in plaque formation, cap stabilization, and lesional inflammation. SMCs demonstrate remarkable heterogeneity and capacity for plasticity both in healthy arteries and during vascular remodeling. A major focus of current research relates to how SMCs expand in the vessel wall. Historically, there has been a long-standing debate regarding their capacity for clonal expansion in humans due to a lack of high-resolution experimental techniques. Recent advances in multi-color lineage tracing mouse models have provided robust evidence supporting the concept that medial SMCs exist in a polyclonal manner, while plaque SMCs expand in a monoclonal or oligoclonal pattern, at least in mice. However, our mechanistic knowledge of how clonal expansion is initiated is still in its infancy. Recent single-cell transcriptomic studies suggested the existence of SMC subpopulations with markedly varying phenotypic profiles. It has therefore been hypothesized that a collection of pre-existing SMC progenitors which are primed to respond to pathological stimuli may exist and form the basis for the emergence of a dominant clone. Alternatively, it is possible that all medial SMCs have the potential to expand, but only a few are ultimately selected due to a survival advantage conferred by somatic mutation, epigenetic alteration, resistance to phagocytic clearance, lateral inhibition, or some other unknown mechanism. Though the molecular drivers underlying SMC clonal expansion remain to be fully elucidated, recent studies have begun to point to some initial clues (21, 43, 44). More work is required to determine whether phenotype modulation happens before or after expansion, the route by which SMCs move along during plaque formation, and if the mechanisms which govern the initiation vs. progression of SMC expansion are distinct. Answering these questions will inform future translational efforts aimed at eliminating or reprogramming subpopulations of clonally expanded SMCs as a means to stabilize plaques and ameliorate atherosclerosis.
